# Current Status of Renal Anemia Pharmacotherapy—What Can We Offer Today

**DOI:** 10.3390/jcm10184149

**Published:** 2021-09-15

**Authors:** Bartłomiej Borawski, Jacek Stanislaw Malyszko, Marlena Kwiatkowska, Jolanta Malyszko

**Affiliations:** 1 Department of Nephrology, Dialysis and Internal Medicine, Medical University of Warsaw, Banacha 1A, 02-097 Warsaw, Poland; bartekborawski@hotmail.com (B.B.); marlenakwiatko@gmail.com (M.K.); 2 1st Department of Nephrology and Transplantology, Medical University of Bialystok, 15-540 Bialystok, Poland; jackmaly@poczta.onet.pl

**Keywords:** anemia, chronic kidney disease, hemodialysis, ESA, iron, hepcidin, HIF inhibitors

## Abstract

Chronic kidney disease (CKD) is one of the fastest-growing major causes of death internationally. Better treatment of CKD and its complications is crucial to reverse this negative trend. Anemia is a frequent complication of CKD and is associated with unfavorable clinical outcomes. It is a devastating complication of progressive kidney disease, that negatively affects also the quality of life. The prevalence of anemia increases in parallel with CKD progression. The aim of this review is to summarize the current knowledge on therapy of renal anemia. Iron therapy, blood transfusions, and erythropoietin stimulating agents are still the mainstay of renal anemia treatment. There are several novel agents on the horizon that might provide therapeutic opportunities in CKD. The potential therapeutic options target the hepcidin–ferroportin axis, which is the master regulator of iron homeostasis, and the BMP-SMAD pathway, which regulates hepcidin expression in the liver. An inhibition of prolyl hydroxylase is a new therapeutic option becoming available for the treatment of anemia in CKD patients. This new class of drugs stimulates the synthesis of endogenous erythropoietin and increases iron availability. We also summarized the effects of prolyl hydroxylase inhibitors on iron parameters, including hepcidin, as their action on the hematological parameters. They could be of particular interest in the out-patient population with CKD and patients with ESA hyporesponsiveness. However, current knowledge is limited and still awaits clinical validation. One should be aware of the potential risks and benefits of novel, sophisticated therapies.

## 1. Introduction

Chronic kidney disease (CKD) is one of the fastest-rising major causes of death internationally, with a global prevalence of 13% [1]. Better treatment of CKD and its complications is crucial to reverse this negative trend. Anemia is a frequent complication of CKD and is associated with unfavorable clinical outcomes [2,3]. The prevalence of renal anemia gradually rises as the estimated glomerular filtration rate (eGFR) decreases [3]. Anemia occurs in approximately half of patients with CKD stage G4 and in more than 90% of the end-stage renal disease patients who undergo dialysis [3,4,5]. Correcting renal anemia can decrease mortality, hospitalization, risk of CKD progression, and improve the health-related quality of life [5,6,7,8,9,10]. The principial mechanism implicated in the development of renal anemia is a combination of inadequate erythropoietin (EPO) synthesis and EPO resistance [11,12]. Other contributing factors include both absolute and functional iron deficiency, chronic inflammation, uremic toxins, disturbed iron homeostasis, shortened red blood cell (RBC) life span, and vitamin deficiencies (vitamin B12 or folic acid) [11,13]. Moreover, hemodialysis itself may contribute to blood loss and damage to RBCs [14]. Screening for and treating anemia is a routine part of the care of CKD patients [15].

At the beginning of the second half of the 20th century, Erslev recognized that plasma from anemic rabbits containing a factor capable of stimulating erythropoiesis could be potentially used as a therapeutic agent [16]. In 1957, researchers revealed that this erythropoietic factor is produced by the kidney [17]. Two decades later, this substance was isolated from urine collected from patients with aplastic anemia and named erythropoietin [18]. Thanks to the advances in biotechnology, the EPO gene was successfully isolated and cloned [19]. This discovery paved the way for the next breakthrough—the development of the recombinant human erythropoietin. The ability to stimulate erythropoiesis with therapeutic agents became a milestone that probably was the greatest breakthrough in nephrology. Before effective treatment for anemia was available, a high proportion of HD patients required regular high-volume blood transfusions with attendant risks of immunological sensitization, iron overload, and viral infections. It should be mentioned that the effects of the blood transfusions were transient, and many patients required chronic repeated transfusional support to alleviate or relieve debilitating symptoms such as exertional shortness of breath, lethargy, and poor physical capacity.

## 2. Erythropoiesis-Stimulating Agents

Before effective treatment for renal anemia became available, a high proportion of hemodialysis patients required blood transfusions with the attendant risks of immunological sensitization, iron overload, and viral infections. Currently, injectable erythropoiesis-stimulating agents (ESAs) with adjuvant iron therapy and/or RBC transfusions represent the mainstay of anemia’s treatment in CKD [20]. ESAs are used for the treatment of CKD anemia that cannot be corrected by iron supplementation alone. The term ESA encompasses short-acting recombinant human erythropoietin (epoetin), medium-acting darbepoetin alfa, long-acting epoetin beta pegol, and their biosimilars [21]. ESAs are characterized by a common mechanism of action but different pharmacokinetic and pharmacodynamic properties [22]. Despite the widespread use of ESAs to treat anemia in CKD, the relative and mortality risks associated with using different types of ESAs still have not been fully elucidated [23]. Sakaguchi et al. concluded that long-acting ESAs might be associated with a higher risk of death among patients undergoing hemodialysis than short-acting ESAs [23]. However, other studies did not confirm these findings [22]. To date, biosimilars have demonstrated comparable safety and effectiveness relative to originator ESAs [24,25]. The selection of the individual therapy depends on the severity of anemia and iron deficiency. 

The development of ESAs has changed the treatment of renal anemia dramatically; however, some concerns have remained, particularly in regard to cardiovascular complications and malignancy. Randomized controlled trials such as CHOIR [26] or CREATE [27] showed that targeting Hb to normal ranges in CKD patients or on HD (Normal Hematocrit Trial) [28] could be harmful and should be avoided. It has led to decreases in achieved Hb levels and increases in RBC transfusions. Furthermore, ESAs hyporesponsiveness and functional iron deficiency represent new clinical concerns to solve. Based on the results of the TREAT trial [29] KDIGO [30] recommended lowering Hb targets in patients with CKD. Data from US Renal Data System (USRDS) and Dialysis Outcomes and Practice Patterns Study (DOPPS) showed clearly that the use of iv iron and blood transfusions increased while the use of ESAs declined [31,32,33]. At present, there are no well-defined guidelines regarding when to initiate ESA treatment in CKD patients.

The Kidney Disease: Improving Global Outcomes (KDIGO) 2012 guidelines recommend starting an ESA on an individual basis [30]. ESAs are administered to most hemodialysis patients with hemoglobin (Hb) <10 g/dL and are not iron deficient [34]. Similarly, KIDIGO anemia guidelines do not indicate a recommended starting dose but state that it should be individualized [30]. The dose of ESA required to reach target Hb varies widely among patients [34]. Generally, the dose is adjusted monthly in response to the Hb (the increase should be in the range of 1–2 g/dL per month) [35]. Either intravenous (iv) or subcutaneous (sc) ESA administration may be used. Several studies suggested that the sc dose of ESA required to achieve a target Hb is approximately 30 percent less than that required with IV administration [25]. SC ESA administration is characterized by slower absorption compared to iv administration, resulting in an extended terminal half-life [36]. In patients receiving ESA, the general Hb target should be individualized to 10–12 g/dL, based upon patient clinical characteristics, symptoms, and preferences. The ESAs effectively increase Hb concentration and improve patients’ quality of life [37,38]. ESAs also act as hepcidin suppressors by inducing erythroferrone (ERFE). Hepcidin, a small polypeptide synthesized by the liver, plays a central role in regulating iron homeostasis by promoting ferroportin’s internalization and degradation, the only known cellular iron exporter [39]. As an effect of hepcidin, extracellular iron decreases via decreased intestinal absorption and iron mobilization from the reticuloendothelial system’s macrophages. Hepcidin levels are frequently elevated in patients with CKD due to inflammatory state, impaired renal clearance, and iron supplementation, contributing to functional iron deficiency and interfering hematopoiesis [37].

There is some evidence that in the case of inflammation (which increases hepcidin) and iron deficiency (which decreases hepcidin), the latter is predominant. This is frequently the case for anemia in CKD. The study of Theurl et al. [40] compared the levels of hepcidin in patients with anemia of chronic disease (ACD), those with iron-deficiency anemia (IDA) or mixed ACD/IDA: hepcidin was increased in patients with ACD compared to control subjects, but in mixed ACD/IDA patients, hepcidin levels were comparable to those observed in IDA patients [41,42].

However, patients treated with ESAs are exposed to supraphysiologic EPO concentrations, potentially leading to adverse events [5,37,43,44]. Some studies have found an increased risk of cardiovascular events and stroke in both hemodialysis-dependent and nonhemodialysis-dependent patients targeted at higher Hb levels [45,46]. Adverse events seem to be related to a high ESA dose rather than with a high Hb target. In this sense, Choukroun et al. [47] in their prospective study showed that a target hemoglobin ≥13 g/dL reduced the progression of chronic allograft nephropathy in kidney transplant recipients without an increase in adverse events. Other adverse effects of ESAs include the development or worsening of hypertension, venous thromboembolism including hemodialysis access, malignancy, and an even greater risk of death [48].

Most of the adverse effects occur when ESAs are used to maintain normal or near-normal Hb as reviewed elegantly by Locatelli et al. [49]. On the other hand, the risk of hypertension appears to be independent of target Hb [50]. Multiple patient safety concerns regarding ESAs therapy safety resulted in a decrease in target Hb. Furthermore, hyporesponsiveness affecting 10% of patients with CKD represents another major clinical challenge in ESAs therapy [51]. Hyporesponsiveness to ESA is defined as a continued need for greater than 300 IU/kg per week erythropoietin or 1.5 mg/kg per week darbepoetin administered SC [52]. Iron deficiency and chronic inflammation are the most important determinants of ESA resistance. In patients with ESA hyporesponsiveness, higher ESA doses are needed to raise Hb levels, likely increasing the risk of cardiovascular events and mortality [46,52]. The lowest ESA dose necessary to achieve the desired Hb level should be used, and excessively high doses in patients with ESA hyporesponsiveness should be avoided. The evidence shows that maintaining adequate iron stores is the most meaningful singular strategy for decreasing ESA requirements and for augmented ESA efficacy. Furthermore, a number of studies have underlined the shortcomings of ESA therapy related to convenience (e.g., mode of administration) [53]. Taken together, these downsides (adverse events, convenience, and hyporesponsiveness) suggest the need for the exploration of novel treatments of CKD anemia.

## 3. Hypoxia-Inducible Factor Inhibitors

Concerns about ESA therapy are a major driver in searching for alternative therapeutic options. Hypoxia-inducible factor prolyl hydroxylase inhibitors (HIF-PHIs) represent a novel approach to the treatment of anemia in patients with CKD [5,12,37,46,54,55,56,57,58,59,60,61,62,63,64,65,66,67,68,69,70]. HIF-PHIs are oral drugs that mimic the natural response to hypoxia independent of cellular oxygen levels [54]. HIF-PHIs undergoing clinical trials include daprodustat, desidustat, enarodustat, molidustat, roxadustat, and vadadustat. Data on selected clinical trials regarding HIF-PHIs are gathered in Table 1 and Table 2 [46,53,66,67,68,69,70,71,72,73,74,75,76,77,78,79,80,81,82,83,84]. HIF-PHIs have multiple targets of action, resulting in the stimulation of endogenous EPO production within the physiological range, suppression of liver hepcidin synthesis, and increased transcription of genes that promote iron utilization, which profoundly differentiates them from presently used ESAs [5,85].

Hypoxia-inducible factor (HIF) is the main transcription factor of hundreds of genes, including the EPO gene expressed in hypoxic tissues and adapted to a hypoxic environment [86]. HIF-PHIs are small molecules that inhibit the prolyl hydroxylase domain (PHD) proteins (PHD1, PHD2, and PHD3), which sense oxygen and control HIF activity [5,87]. HIF-PHIs by decreasing prolyl hydroxylase activity lead to the stabilization of HIF-α in the liver and kidney, resulting in upregulation of the endogenous EPO [46]. HIF is a heterodimer complex comprised of an oxygen-sensitive α-subunit (either HIF-1α, HIF-2α, or HIF-3α), which is rapidly degraded by PHD in the presence of oxygen, and a constitutively expressed β-subunit [71]. HIF-PHIs differ in molecular structure and likely have different levels of selectivity for the three main PHD isoforms [5]. Moreover, animal studies have shown that HIF also controls the expression of proteins playing a role in iron metabolism and utilization (upregulation of transferrin, soluble transferrin receptor 1 (sTfr1), ceruloplasmin, divalent metal transporter 1 (DMT1), duodenal cytochrome b (Dcytb), and downregulation of hepcidin) [37,88]. It is widely accepted that EPO production and iron-regulating genes are mainly HIF-2α controlled, with HIF-1α playing a smaller role [5,88,89]. Increases in soluble transferrin receptor 1 improve iron availability, given its role as a carrier protein for transferrin required to import iron into the cell [53]. HIF-2α directly upregulates ferroportin and indirectly inhibits hepcidin expression promoting iron availability [52,90]. Supporting this notion, a meta-analysis performed by Wen et al. showed HIF-PHIs-related decreases in serum hepcidin and ferritin, together with increases in transferrin and TIBC, suggesting an improvement in iron utilization compared to the standard therapy with ESA and IV iron [37]. The administration of HIF-PHIs to patients with CKD was consistently associated with decreased plasma hepcidin levels in phase 2 and phase 3 clinical trials [40,42,46,53,66,67,68,69,70,71,72,73,74,75,76,77,78,79,80,81,82,83,84]. Researchers suggest that hepcidin suppression most likely results from the stimulation of erythropoiesis, as it seems that hepcidin is not a direct transcriptional target of HIF. There is no research report whether HIF can affect the expression of hepcidin gene, and there may be other mechanisms between HIF and hepcidin. Although ESAs were also reported to suppress hepcidin production through erythroferrone upregulation, Akizawa et al. observed a trend toward a decrease in hepcidin, following the switching from an ESA to enarodustat [46]. It is anticipated that the latter most likely results from differences in effectiveness in promoting erythropoiesis and EPO-mediated hepcidin regulators such as ERFE; however, future studies are needed to provide an answer.

All published phase II trials indicate that HIF-PHI therapy is at least as efficacious as conventional ESA therapy in managing Hb levels in both nondialysis-dependent CKD (ND-CKD and dialysis-dependent (DD-CKD) patients [55,56,57,58,59,60,61,62,63,64,65,66,67,68,91,92,93,94,95,96,97,98]. The meta-analysis of 26 randomized controlled trials involving 2804 patients with CKD comparing the use of HIF-PHIs versus ESAs or placebo in the treatment of renal anemia found HIF-PHIs superior to placebo and at least as efficacious as classic ESAs in the short term [5]. Likewise, a meta-analysis by Qie et al., including 1010 patients, showed that roxadustat has a higher mean Hb level than placebo or EPO [91]. HIF-PHIs have demonstrated efficacy and safety in phase 2 and 3 trials in several different cohorts of CKD patients with anemia who are either ND-CKD or DD. Wang et al. reported that among the included HIF-PHIs, roxadustat was associated with a favored effect on Hb compared to classic ESAs [5]. Roxadustat administration leads to increases in endogenous EPO levels, with peak increases 8–12 h postdose in healthy volunteers and patients with CKD [61,62,63,64,65] With a half-life of 10 h, roxadustat is administered three times weekly [91]. HIF-PHIs such as roxadustat offer the promise of decreased inflammation, a better use of existing iron stores, and an enhanced gastrointestinal absorption of oral iron, along with an acceptable safety profile. In a European, multicenter, double-blind trial in a predialysis patient population, the safety profile of roxadustat was generally comparable with placebo [53]. Moreover, in roxadustat-treated patients, the markers of both iron (increased iron levels, increased transferrin levels, and total iron-binding capacity-TIBC, decreased hepcidin) and lipid (reduced low-density lipoprotein) metabolism were improved. It is suggested that the beneficial effect of roxadustat on lipid metabolism is primarily mediated by HIF-dependent effects on acetyl coenzyme A (required for cholesterol synthesis) and on the degradation of 3-hydroxy-3-methylglutaryl coenzyme A reductase (a rate-limiting enzyme in cholesterol synthesis). Roxadustat-related decreases in hepcidin and increases in soluble transferrin receptor levels have been observed in many studies [61,62,63,64,65]. In addition, recent phase 3 studies demonstrated that roxadustat was superior to placebo and noninferior to standard ESAs to treat CKD-related anemia [53,56,74,75,76,77,78,79,80,81]. Patients who newly initiate dialysis require the highest doses of ESAs and have the greatest risk for mortality during the first year on dialysis. 

Regarding safety, HIFs also activate, additionally to EPO, a large number of genes, which can also cause beneficial effects. Potential clinical benefits of HIF-PHIs therapy include: achieving Hb target with a 5–17-fold lower plasma EPO levels relative to those receiving ESA; and a lipid-lowering effect, blood-pressure reduction, anti-inflammatory effects, protection from ischemic injuries, and protective effects with regard to CKD [5,92,93,94,95,96,97,98]. Roxadustat and daprodustat are demonstrated to reduce triglyceride, total cholesterol, and low-density lipoprotein levels [62,63,69,71]. In animal models, HIF-PHIs have been shown to lower blood pressure; however, clinical trials did not confirm this finding [46,53,56,58,66,67,68,69,70,71,72,73,74,75,76,77,78,79,80,81,82,83,84]. A growing body of evidence suggests that HIF-PHIs increase Hb levels, with EPO levels remaining within the physiological range [46]. Studies in ND-CKD and DD-CKD patients showed that HIF-PHIs administration was associated with much lower plasma EPO increases than recombinant human EPO [71]. In addition, no accumulation of EPO was reported after a repeated dosing of enarodustat [32]. Since HIF transcription factors control or interact with many biologic processes, there is a concern about nonerythropoietic adverse effects. The areas of concern regarding HIF-PHIs include potential tumor-promoting effects, thrombosis, cardiovascular disease, and the progression of diabetic retinopathy [85,86,87,88,89]. To date, the most frequently reported AEs in undergoing clinical trials were gastrointestinal disorders (nausea, diarrhea, and vomiting) [5,12]. In short-term studies, no serious adverse events attributed to HIF-PHIs were observed, although the clinical prevalence of adverse events with long-term HIF-PHIs treatment needs to be determined. The occurrence of AEs is likely to depend on the pharmacokinetics and dosing of HIF-PHIs. For example, statistically significant rises in plasma vascular endothelial growth factor (VEGF) levels were observed when relatively high doses of daprodustat (50–100 mg) were given to healthy subjects but were not detected with lower doses sufficient to maintain Hb in CKD patients. Recently, Chertow et al. [96] reported that vadadustat, as compared with darbepoetin alfa, met the prespecified noninferiority criterion for hematologic efficacy but not the prespecified noninferiority criterion for cardiovascular safety in patients with ND-CKD, which was a composite of death from any cause, nonfatal myocardial infarction, or nonfatal stroke. On the other hand, Provenzano et al. [99] evaluated the efficacy and cardiovascular safety of roxadustat versus placebo by analyzing data pooled from three phase 3 studies of roxadustat in patients with nondialysis-dependent CKD and CKD-related anemia. They found that there were no increased risks of MACE (HR, 1.10; 95% CI, 0.96 to 1.27), MACE + (HR, 1.07; 95% CI, 0.94 to 1.21), all-cause mortality (HR, 1.08; 95% CI, 0.93 to 1.26), or individual MACE+ components in patients treated with roxadustat versus those treated with placebo. 

**Figure 1:**
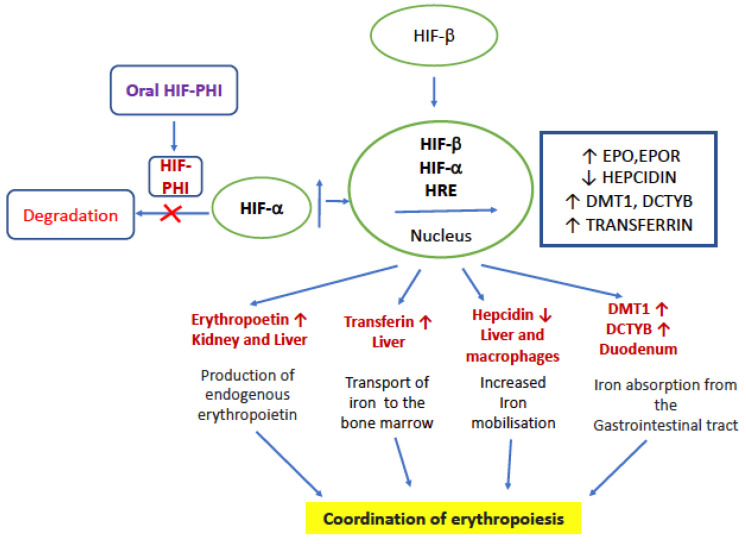
Mode of action of HIF-PHI. EPO—erythropoietin, EPOR—erythropoietin receptor, DCYTB, duodenal cytochrome B; DMT1, divalent metal transporter, HRE—hypoxia responsive element, and HIF—hypoxia inducible factor.

Taken together, HIF-PHIs can reduce the degradation of HIF irrespective of oxygen levels and decrease the production of hepcidin, thereby increasing the EPO level, utilization of iron, and stimulating the production of erythrocytes/hematopoiesis [5,37]. The mode of action is given in Figure 1. HIF-PHIs effectively decrease the need for iron replacement therapy and are effective in patients with ESA hyporesponsiveness caused by inflammation. Traditional ESAs for patients with ND-CKD require injection and medical visits, while HIF-PHIs have the advantage of oral administration. These benefits include convenience, simpler production, and pain avoidance.

Roxadustat is approved in Japan and China. On 24 June 2021, the (CHMP) adopted a positive opinion, recommending the granting of a marketing authorization for the medicinal product Evrenzo, intended for the treatment of anemia symptoms in patients with chronic kidney disease (EMA/CHMP/341057/2021) [100]. However, on 15 July the Cardiovascular and Renal Drugs Advisory Committee of the FDA voted against approval of roxadustat for the treatment of anemia due to chronic kidney disease for patients not on dialysis and on dialysis due to the higher portion of ND-CKD patients and DD-CKD patients experienced vascular access thrombosis compared with controls [101,102,103]. Table 3 summarizes the potential advantages and disadvantages of HIF inhibitors [5,104,105,106,107,108,109,110,111].

## 4. Iron Supplementation

Good iron metabolism is essential for the efficacy of ESAs and HIF-PHIs [55]. Inflammation and iron depletion are the most important causes of hyporesponsiveness to ESA treatment [116]. Dysregulation of iron homeostasis in patients with CKD is multifactorial, including diminished renal clearance of hepcidin, inflammation, absolute iron deficiency, and functional iron deficiency [11,13]. Absolute iron deficiency is defined by severely decreased total iron stores. On the other hand, functional iron deficiency is a state of adequate iron stores but decreased iron availability for erythropoiesis [15,117,118]. A combination of both absolute and functional iron deficiency may also be present, suppressing erythropoiesis at the iron-dependent stage of Hb synthesis. Based on expert opinion, absolute iron deficiency is diagnosed when transferrin saturation (TSAT) is ≤20% and the serum ferritin concentration ≤ 100 ng/mL in nonhemodialysis patients and ≤200 ng/mL among hemodialysis patients [119,120]. Functional iron deficiency is characterized by TSAT ≤ 20% and elevated ferritin levels [119,120]. The KDIGO 2012 Anemia Guidelines recommend the regular monitoring of iron status (TSAT and ferritin at least every 3 months) during ESA therapy and before deciding to start or continue iron therapy [30]. Since iron deficiency plays an important role in anemia in CKD, iron agents are the mainstay of therapy for renal anemia. Iron therapy is introduced to replenish iron stores, increase Hb level to the desired level, and decrease needs for ESA. According to the guidelines, adult CKD patients with anemia or on ESA therapy should receive a trial of iv iron (or a 1–3 month trial of oral iron for ND-CKD patients) if an increase in Hb concentration or a decrease in ESA dose is desired and TSAT is <30% and ferritin is <500 µg/L [22]. The continuation of iron therapy should be based on an integrated assessment of Hb responses, iron status tests, ESA dose/responsiveness, ongoing blood losses, and clinical status, although the available data were considered insufficient for recommending long-term iv dosing strategies [30]. Carboxymaltose iron- FCM, isomaltoside iron, and ferumoxytol are among new iv iron preparations. Results from a prospective, multicentric, randomized controlled study of more than 2000 patients undergoing hemodialysis (HD) support the efficacy and safety of relatively high-dose iv iron therapy among hemodialysis-dependent patients treated with ESA [20,121]. Monthly administration of 400 mg iv iron in patients with serum ferritin < 700 µg/L and TSAT ≤ 40% decreases ESA use and lowers the risk of all-cause death, nonfatal myocardial infarction, nonfatal stroke, and hospitalization for heart failure compared with iv iron administered in a reactive fashion for ferritin < 200 µg/L or TSAT < 20% [20,121]. FIND-CKD study in ND-CKD patients demonstrated that iv iron dosed to target ferritin of 400–600 µg/L compared with oral iron quickly reached and maintained Hb. Moreover, iv iron dosed to a target ferritin of 400–600 µg/L was superior to iv iron dosed to target ferritin of 100–200 µg/L for achieving a Hb increase of ≥ 1 g/dL [122]. The possible negative effects of iron compounds are still a matter of debate. Given its ability to participate in Fenton reaction, excessive iron supplementation may promote oxidative stress and potentially contribute to cardiovascular disease risk, CKD progression, and other organ damage in CKD patients [5,20]. In addition, iron is an essential trace metal for nearly all infectious microorganisms; thus, it has been hypothesized that excessive iron supplementation may lead to a permissive environment for infectious processes. Thus, the KDIGO workgroup recommends withholding iv iron during active infections [30].

Recently, a meta-analysis of epidemiological studies and randomized controlled trials in DD-CKD patients did not show any significant rise in cardiovascular events or infections in the group receiving a high dose of iron when compared to the low-dose group [123]. Likewise, a secondary analysis of PIVOTAL demonstrated that the risks of infections were similar between high-dose and low-dose iv iron groups [124]. However, a study by Li and colleagues showed that more intensive iv iron administration in HD-CKD patients is associated with a higher risk of mortality and infection-related events [125]. Whether high-dose-iron administration increases rates of infections or cardiovascular events in ND-CKD patients is still conflicting and remains to be studied. Considering the areas of uncertainty, caution is still warranted regarding more aggressive iv iron strategies in CKD patients, until more data are available. While there is general agreement that iv iron supplementation is the suitable method for DD-CKD patients, either iv or oral iron can be given to ND-CKD patients [20]. The KDIGO recommendation to use iv rather than oral iron in DD-CKD patients was supported by a number of clinical studies that demonstrated a greater Hb increase with iv iron than oral iron [30].

In addition, follow-up versions of iron sucrose have emerged to treat iron deficiency anemia, including renal anemia, in a number of countries worldwide. The assumption was that iron sucrose similar agents could be considered therapeutically equivalent to the originator iron sucrose. That said, Rottembourg et al. demonstrated that the switch from the originator iron sucrose to an iron sucrose similar agent resulted in destabilization of a well-controlled population of dialyzed patients and led to an increase in total anemia drug costs, putting a question mark on their equivalence [126].

Currently available oral iron agents have variable effectiveness in increasing Hb, ferritin, and TSAT, as well as in reducing the use of ESAs or blood transfusions [127]. Compared with iv iron, oral iron is less effective in correcting iron deficiency and reducing ESA needs [128]. Given that gastrointestinal side effects are frequent with oral iron administration, many oral iron preparations are poorly tolerated. Additional concerns include poor absorption and altering colonic microflora. Considering that oral iron formulations are noninvasive, avoid injection site complications, and consume venous capital for future access, creation can be a suitable method for ND-CKD patients. Furthermore, it should be underlined that most of the data were derived from comparing older iron preparations. In addition to traditional iron agents, novel oral iron preparations have been developed to improve the treatment efficacy. One of these agents, ferric citrate, has been shown to improve iron parameters and has been proven to be an effective treatment for anemia in ND-CKD patients [129,130]. Furthermore, ferric citrate has been proven effective in reducing iv iron and ESA needs, with a good tolerability, in patients undergoing HD [131]. The liposomal iron represents another example of a new generation of oral iron preparations, which shows high gastrointestinal absorption and high bioavailability [132]. Compared with other traditional oral iron agents, liposomal iron avoids the direct contact of iron with the intestinal mucosa and bypasses the intestinal hepcidin–ferroportin block via a different uptake mechanism. In a small trial by Pisani et al., liposomal iron was proven to increase Hb in ND-CKD patients [133]. Ferric pyrophosphate citrate (FPC) is a water-soluble iron salt that can be administered via dialysate or iv [130]. Compared with iv iron agents, FPC via dialysate presents the advantage of supplying the iron directly to transferrin. FPC has been proven effective in Hb maintaining and decreasing ESA needs [134]. Iv iron agents have comparable efficacy in improving Hb, ferritin, and TSAT and reducing the use of ESAs or blood transfusions [135]. However, some data show that iron sucrose similars have decreased efficacy and safety compared with parent iron sucrose [135]. Kalra et al. [136] studied 351 iron-deficient ND-CKD patients and randomized them 2:1 to either IV iron isomaltoside 1000 or iron sulphate (100 mg of elemental oral iron twice daily for 8 weeks). Isomaltoside 1000 was superior to oral iron sulphate in increasing Hb levels and was well tolerated. Results from the PIVOTAL trial [20,137,138] are reassuring, regarding the iv iron administration in patients with short dialysis vintage, no or minimal inflammation, and up to ferritin of 700 ng/mL. However, the question arises, whether this regimen is safe and efficacious in patients with comorbidities, inflamed, and dialyzed for a long-time. In the modern world, this is a prevalent population in HD units. In ND-CKD patients, FIND-CKD study ferritin levels of 800 ng/mL or above did not result in an increase in serious adverse events after administration of ferric carboxymaltose [122]. In addition, the grey area of knowledge is the potential mechanisms by which a high-dose iron regimen (iron sucrose) was associated with improved outcomes in the PIVOTAL study [20]. Macdougall et al. also showed a randomized, controlled trial, that ferumoxytol and iron sucrose showed comparable efficacy and adverse events rates [139]. In addition, recently, Macdougall et al. [140] reported that long-term administration of ferumoxytol has noninferior efficacy and a similar safety profile to iron sucrose when used to treat IDA in patients with CKD undergoing hemodialysis. Whether newer iron preparations such as ferric carboxymaltose or iron isomaltose yield the same positive results remains to be studied. Hypophosphataemia is an increasingly recognized side effect of ferric car-boxymaltose (FCM) and possibly iron isomaltoside/ferric derisomaltose (IIM), which are used to treat iron deficiency. Schaefer et al. [141] included 42 clinical trials in the meta-analysis and found that FCM induced a significantly higher incidence of hypophosphataemia than IIM (47%, 95% CI 36–58% versus 4%, 95% CI 2–5%) and significantly greater mean decreases in serum phosphate (0.40 versus 0.06 mmol/L). More severe iron deficiency and normal kidney function are risk factors for hypophosphataemia. However, this side effect seems less important in CKD patients who have often high basal phosphate levels. Future trials focusing on long-term effects and optimal dosing strategies of novel oral iron preparations should be conducted.

## 5. Other New Therapeutic Strategies

As previously discussed, iron metabolism is dysregulated in patients with CKD, contributing to the development and progression of CKD-related anemia. Given that hepcidin is a key regulator of iron balance, the strategy of decreasing hepcidin was recently proposed as a promising therapeutic option. These findings lead to the exploration of therapeutic approaches of targeting iron metabolism, including inhibitors of hepcidin, IL-6 antibodies, and other anti-inflammatory biologicals (inhibition of the BMP6, SMAD, HJV, and IL-6/STAT cascades) [142]. Hepcidin antibodies have been shown to bind human (and monkey) hepcidin, inhibit its action on ferroportin, enhance the dietary iron absorption, and promote the mobilization of its stores [142]. The ferroportin stabilizers tend to reduce hepcidin expression, inhibit its action, and prevent ferroportin degradation [142]. Antiferroportin monoclonal antibody (LY 2928057) blocks hepcidin interaction with its receptor, thereby reducing ferroportin internalization and allowing more iron flux [138]. In CKD patients, the administration of LY2928057 resulted in an increase in Hb and a reduction in serum ferritin levels [53]. Interleukin-6 (IL-6) is a major inducer of hepcidin production. A small study demonstrated that inhibitors of the IL-6 pathway decreased hepcidin levels and ameliorated anemia in Castleman’s disease. Likewise, blocking antibodies to the IL-6 ligand has been proven effective in lowering hepcidin [142]. Siltuximab, the anti-IL-6 chimeric monoclonal antibody, increased Hb levels by 2.1 g/dL; however, it has been associated with a greater risk of infections [143]. Lipocalins are low-molecular-weight proteins that naturally bind, store, and transport a wide spectrum of molecules, e.g., hormones [144]. Anticalins are proteins with engineered ligand-binding properties derived from the human’s lipocalin. PRS-080, an anticalin against hepcidin, efficiently and specifically sequester hepcidin [144,145]. A study in DD-CKD patients demonstrated neutralization of hepcidin by PRS-080 and a subsequent increase in serum iron and TSAT following PRS-080 administration [144,145].

## 6. Conclusions

CKD is one of the main causes of death internationally and requires the better prevention and treatment. For years, ESAs have remained a cornerstone of therapy for anemia in CKD together with iron supplementation. Novel therapeutic strategies are imperative for improving anemia in patients with CKD. HIF-PHIs, unlike conventional ESAs, stimulate the transcription of the EPO gene in the kidneys and liver, leading to increased levels of endogenous erythropoietin. HIF-PHIs have been reported to be at least as efficacious as ESAs in the correction of anemia in CKD, without increasing the incidence of adverse events in the short term. Furthermore, HIF-PHIs seem to offer unique pharmacological effects such as improving iron utilization and pleiotropic effects. On the other hand, classic ESAs have a comprehensive safety profile, which should be the future comparison standard for the new class of drugs.

## 7. Summary

For almost 40 years, we have expanded our understanding of ESAs and iron (for iron even more) as major antianemic drugs. As the first of innovative ESAs started to come off patent, biosimilar biologic agents have been introduced as alternatives for the treatment of anemia in many countries [146,147]. In addition, follow-up versions of iron sucrose have emerged to treat iron-deficiency anemia, including renal anemia, in a number of countries worldwide. The assumption was that iron sucrose similar agents could be considered therapeutically equivalent to the originator iron sucrose. Many promising novel agents appeared in recent years; however, some of them such as peginesatide turned out to be a falling star. In the 21st century, anemia still remains a widespread complication of CKD that contributes to worse outcomes and lowers the quality of life. Taking into consideration the fact that blood products are not only a scarce resource but they are often in a short supply, we should also be concerned about the low but still real possibility of viral transmission and the increased risk of HLA sensitization, which may be responsible for longer time on the waiting list for transplantation, increased graft rejection, and poorer graft survival [148]. In addition, the usage of iv iron agents and multiple blood transfusions that inevitably result in cumulative iron overload represented another clinical problem with unknown long-term clinical relevance [149]. These trends highlight the need for new research to find agents effective in these new settings with no safety concerns.

**Figure 2:**
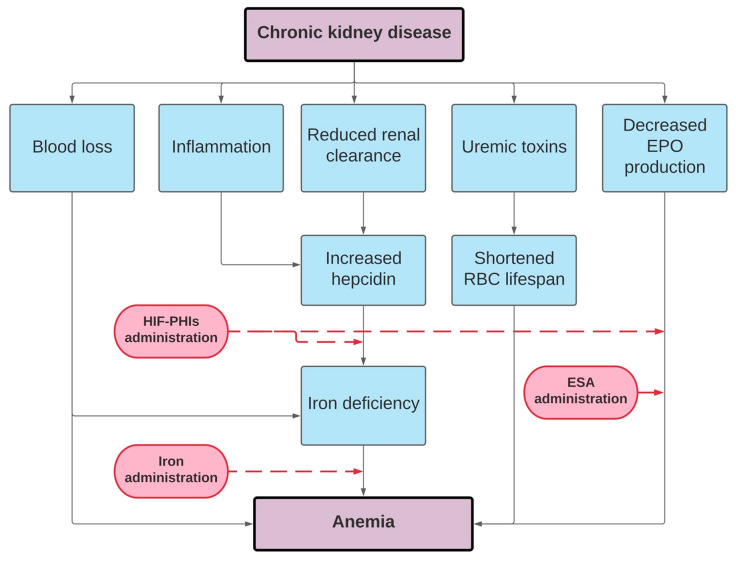
Pathophysiology of renal anemia and treatment options.

Data from observational, randomized controlled trials and case reports lead to the exploration of therapeutic approaches for treating the anemia of CKD. Inhibitors of the hepcidin–ferroportin axis are in development at preclinical and clinical stages. Several HIF stabilizers are studied in phase 2 and 3 trials with promising results. HIF stabilizers have been shown to be at least as efficacious as classic ESA in the short term. The ultimate goal of renal anemia treatment is to affect iron together or without “good, old” strategies such as ESAs therapy or “novel and hopefully better” such as HIF stabilizers (Figure 2). HIF-PHIs could hold the potential to change the scene of renal anemia treatment in the near future, if they demonstrate a noninferior safety profile compared to classic ESAs. Renal anemia treatment with a particular focus on iron was the matter of “Optimal Anemia Management: Conclusions from KDIGO Conference in December 2019” in the recent publication in 2021 [150]. We did explore new and ongoing controversies, together with possible implications for changes of the current KDIGO anemia guideline, and proposals of research agenda. The second controversy conference is planned for the end of 2021 with a particular focus on issues more specifically related to HIF inhibitors.

## Figures and Tables

**Table 1:** HIF stabilizers—data on published peer-reviewed phase II studies in dialysis-dependent CKD and nondialysis-dependent CKD.

Compound	Dose	Study Population	Duration (Weeks)	Comparator	Main Finding	Ferritin	TIBC	Hepcidin	Additional Finding	Reference
Roxadustat	0.7–2.0 mg/kg body weight; thrice weekly	116 ND-CKD patients	4	placebo	dose-dependent increases in blood Hb	↓	↑*	↓*	Adverse events were similar roxadustat versus placebo	Besarab et al. [61]
	50–150 mg; thrice weekly	145 ND-CKD patients	16–24	-	92% of patients achieved Hb response	↓*	↑*	↓*	Total cholesterol level was reduced	Provenzano et al. [62]
	1.1–2.25 mg/kg body weight; thrice weekly	91 ND-CKD patients	6	placebo	Hb increase ≥1 g/dL from baseline was achieved in 80% of subjects in the low-dose cohort and 87.1% in the high-dose cohort	↓*	↑*	↓*	significant reductions in cholesterol were noted	Chen et al. [63]
	1.0–2.0 mg/kg; thrice weekly	54 DD-CKD patients (part 1) 90 DD-CKD patients (part 2)	6 (part1) 19 (part 2)	rHuEPO	Roxadustat was well tolerated and effectively maintained Hb levels	↓	↑	↓*	Hepcidin level reduction was greater at roxadustat 2.0 mg/kg versus epoetin alfa	Provenzano et al. [64]
	1.0–2.5 mg/kg; thrice weekly	60 DD-CKD patients	12	-	Roxadustat increased mean Hb and reduced hepcidin levels	↓*	↑*	↓*	A greater Hb response in the cohorts receiving iron compared with no-iron cohort	Besarab et al. [65]
	1.1–2.3 mg/kg; thrice weekly	87 DD-CKD patients	6	rHuEPO	59.1%, 88.9%, and 100% of the low-, medium-, and high-dose subjects respectively, maintained their Hb levels after 5- and 6-weeks versus 50% of the epoetin alfa-treated subjects	↓	↑*	↓	Significant reductions in cholesterol. Hepcidin levels reduced significantly dose-dependent manner in the highest dose group versus epoetin alfa-treated group	Chen et al. [63]
		324 ND-CKD	24	Darbepoetin alfa	Confirmation the noninferiority of roxadustat to darbepoetin alfa	↔	↔	↔	Roxadustat doses required to maintain target Hb levels did not appear to be influenced by hs-CRP as with darbepoetin-treated patients with high hs-CRP	Akizawa et al. [80]
	70 or 100 mg thrice weekly	916 ND-CKD	240 (4.5 years)	Placebo (2:1)	hemoglobin mean (SD) change from baseline over weeks 28–52 was significantly larger for roxadustat (2.00 [0.95]) versus placebo (0.16 [0.90])	↓*	↑*	↓*	Roxadustat lowered mean LDL cholesterol. There was no significant between-group difference in progression of CKD	Coyne et al. [81]
	70 or 100 mg thrice weekly	616 ND-CKD	104	Darbepoetin alfa	Hemoglobin response with roxadustat was noninferior to DA (roxadustat: 256/286, 89.5% versus DA: 213/273, 78.0%, difference 11.51%, 95% confidence interval, 5.66–17.36%)	↓	↑	Not available	There was no difference between groups regarding the composite endpoints major adverse cardiovascular events (MACE) and MACE +	Barratt et al. [94]
	70, 100, 150, or 200 mg TIW	741 DD-CKD (including D)	52	Epoetin alfa	Roxadustat was noninferior (least squares mean difference: 0.48 (95% confidence interval: 0.37, 0.59); *p* < 0.001) to epoetin alfa	↓	↑*	↓	In regard to blood transfusion, roxadustat was noninferior to epoetin alfa	Charytan et al. [95]
Vadadustat	450 mg; once daily	210 ND-CKD	20	placebo	The primary endpoint (mean Hb level of 11.0 g/dL or more or a mean increase in Hb of 1.2 g/dL) was met in 54.9% of patients on vadadustat and 10.3% of patients on placebo	↓*	↑*	↓*	Significant increases in both reticulocytes and TIBC and significant decreases in both serum hepcidin and ferritin levels	Pergola et al. [66]
	240, 370, 500, or 630 mg; once daily	93 ND-CKD	6	placebo	Compared with placebo, vadadustat significantly increased Hb after 6 weeks in a dose-dependent manner	↓*	↑*	↓*	Vadadustat increased the TIBC and decreased concentrations of ferritin and hepcidin. No significant changes in blood pressure, vascular endothelial growth factor, C-reactive protein, or total cholesterol	Martin et al. [67]
	300 mg once daily or 450 mg once daily or 450 mg thrice weekly	94 DD-CKD	16	Epoetin	Vadadustat maintained mean Hb concentrations in subjects on hemodialysis previously receiving epoetin	↓	↑*	↓*	The most frequently reported AEs were nausea (11.7%), diarrhea (10.6%), and vomiting (9.6%)	Haase et al. [68]
Daprodustat	0.5, 2, or 5 mg once-daily	72 ND-CKD patients	4	placebo	Daprodustat produced dose-dependent effects on Hb, with the highest dose resulting in a mean increase of 1 g/dL at week 4	↓	↑	↓	No clinically significant elevations in plasma vascular endothelial growth factor concentrations were observed	Holdstock et al. [69]
	10, 25, 50, or 100 mg once daily	70 ND-CKD patients	4	placebo	Daprodustat produced dose-dependent increase in EPO concentrations and consequent increases in reticulocytes and Hb levels	↓	↑	↓	A dose-dependent decrease in hepcidin levels and increase in total and unsaturated iron binding were observed in all daprodustat-treated patients	Brigandi et al. [70]
	0.5, 2, or 5 mg once-daily	82 DD-CKD patients	4	rHuEPO	Treatment with daprodustat in the 5 mg arm maintained mean Hb concentrations after the switch from recombinant human erythropoietin, whereas mean Hb decreased in the lower-dose arms	↓	↑	↔ (5 mg)	No clinically significant elevations in plasma vascular endothelial growth factor concentrations were observed	Holdstock et al. [69]
	10, 25, 50, or 100 mg once daily	83 DD-CKD patients	4	placebo	Daprodustat produced a dose-dependent increase in EPO concentrations and consequent increases in reticulocytes and Hb levels	↓	↑	↓ (10 and 25 mg)	A dose-dependent decrease in hepcidin levels and increase in total and unsaturated iron binding were observed in all daprodustat-treated patients	Brigandi et al. [70]
	4, 6, 8, or 10 mg once daily	97 DD-CKD patients	4	placebo	Daprodustat produced dose-dependent increase in Hb relative to placebo	↓	↑	↓	The doses evaluated in the study have moderately increased endogenous EPO without changes in circulating VEGF levels	Akizawa et al. [71]
	4, 6, 8, 10 or 12 mg once daily	216 DD-CKD patients	24	placebo (4 weeks then rHuEPO)	Daprodustat produced dose-dependent changes in Hb over the first weeks after switching from a stable dose of rhEPO as well as maintained Hb target levels over 24 weeks	↓	↑	↓	Daprodustat demonstrated an adverse event profile consistent with the HD population	Meadowcroft et al. [72]
Molidustat	25, 50, and 75 mg once daily; or 25 and 50 mg twice daily	121 ND-CKD patients	16	placebo	Molidustat treatment was associated with estimated increases in mean Hb levels of 1.4–2.0 g/dL	↓	↔	↓	No changes in cholesterol levels were observed	Macdougall et al. [58]
	25–75 mg daily	124 ND-CKD patients	16	Darbepoetin alfa	Hb levels were maintained within the target range after switching to molidustat	↓	↔	↓	No changes in cholesterol levels were observed	Macdougall et al. [58]
	25–150 mg daily	199 HD—CKD patients	16	rHuEPO	Hb levels were maintained within the target range after switching to molidustat 75 and 150 mg	↔	↔	↔	No changes in cholesterol levels were observed	Macdougall et al. [58]
	75 mg daily	51 PD	36		The mean Hb level was maintained in the target range of ≥11.0 and <13.0 g/dL during the evaluation period and more broadly from Week 12 to Week 36	↓*	↑*	↓*	The responder rate (95% CI) during the evaluation period was 54.9% (40.3, 68.9)	Akizawa et al. [97]
Enarodustat	2, 4, or 6 mg once daily	94 corr 107 conv ND-CKD	30	placebo (first 6 weeks)	The proportion of subjects in the conversion group who maintained Hb levels within ±1.0 g/dL of baseline did not differ between each enarodustat arm and placebo arm during Period 1.	↓*	↑*	↓*	Enarodustat was associated with decreases in hepcidin and ferritin and increased TIBC and was generally well tolerated	Akizawa et al. [46]
		216 ND-CKD	24	darbepoetin	The mean Hb level during the evaluation period in the enarodustat arm was 10.96 g/dL (95% confidence interval [CI]: 10.84 to 11.07 g/dL) with a difference of 0.09 g/dL (95% CI: −0.07 to 0.26 g/dL) between arms, establishing its noninferiority to darepoetin	↓*	↑*	↓*	No apparent differences in the incidence of adverse events between arms	Akizawa et al. [97]
Desidustat	100, 150, 200 mg, every alternate day	117 ND-CKD	6	placebo	There was dose-related increase in Hb across all doses compared to placebo	↓*	↑*	↓*	There was no significant change in vital signs, electrocardiographic parameters, or safety laboratory values	Parmar et al. [73]

↑*—a significant increase, ↑ nonsignificant increase, ↓* significant decrease, ↓ nonsignificant decrease, ↔ no change—none, ND-CKD—nondialysis-dependent chronic kidney disease, DD-CKD—dialysis-dependent chronic kidney disease, kidney disease, rHuEPO—recombinant human erythropoietin.

**Table 2:** The main results of selected phase 3 studies of HIF-PIHs in CKD patients.

Compound	Study Design and Population	Duration (Weeks)	Most Important Findings	Ref.
Roxadustat	154 ND-CKD patients received roxadustat or placebo	8	Roxadustat group had a higher mean Hb level than those in the placebo group Roxadustat was associated with reduced levels of hepcidin and cholesterol	Chen et al. [74]
	305 HD-CKD patients undergoing ESA therapy for at least 6 weeks received roxadustat or epoetin afla	26	Roxadustat was noninferior to epoetin alfa The decrease in total cholesterol was greater with roxadustat than with epoetin alfa	Chen et al. [75]
	56 PD-CKD patients (43 ESA converted and 13 ESA-naïve) received roxadustat.	24	Roxadustat at all tested doses has been proven effective in maintaining Hb levels in PD-CKD patients	Akizawa et al. [76]
	30 HD-CKD patients; switch from darbepoetin to roxadustat	4	Roxadustat enhances hematopoiesis and improves iron metabolism early after switching treatments, resulting in increased iron consumption compared with patients who continued darbepoetin	Ogawa et al. [56]
	2781 ND-CKD patients received roxadustat or placebo	28–52	Roxadustat effectively increased Hb and reduced the need for red blood cell transfusion Adverse event profile of roxadustat was comparable to that of placebo	Fishbane et al. [77]
	594 ND-CKD patients received roxadustat or placebo	28–52	Roxadustat demonstrated superior efficacy versus placebo both in terms of Hb response rate and change in Hb from baseline. The safety profiles of roxadustat and placebo were comparable.	Shutov et al. [37]
	1043 HD-CKD patients received roxadustat or epoetin alfa	28–52	Roxadustat was noninferior to epoetin alfa in correcting and maintaining Hb levels	Provenzano et al. [78]
	303 HD-CKD patients received roxadustat or darbepoetin alfa	24	Roxadustat was noninferior to darbepoetin alfa	Akizawa et al. [79]
	99 ESA-naïve, partially iron-depleted NDD-CKD patients received roxadustat at dose 50 or 70 mg	18–24	Roxadustat increased and maintained Hb	Akizawa et al. [80]
	922 ND-CKD patients received roxadustat or placebo	28–52	Roxadustat demonstrated superior efficacy versus placebo in terms of Hb response rate and change in Hb from baseline	Coyne et al. [81]
	616 ND-CKD patients received roxadustat or darbepoetin alfa	104	Roxadustat was noninferior to darbepoetin alfa and maintained HB up to 2 years	Barratt et al. [94]
	4277 ND-CKD received Roxadustat or placebo	52	Roxadustat was more effective than placebo at increasing hemoglobin in ND-CKD patients with anemia, decreased transfusion rate and was noninferior to placebo with respect to risk of MACE	Provenzano et al. [99]
Vadadustat	255 HD-CKD patients received vadadustat and darbepoetin alfa	52	Vadadustat was as well tolerated and effective as darbepoetin alfa in maintaining Hb levels within the target range	Nangaku et al. [82]
	1751 patients with ESA-untreated ND-CKD and 1725 with ESA-treated ND-CKD	52	Vadadustat, as compared with darbepoetin alfa, met the prespecified noninferiority criterion for hematologic efficacy but not the prespecified noninferiority criterion for cardiovascular safety in patients with ND-CKD	Chertow et al. [96]
Daprodustat	28 HD-CKD patients received daprodustat	24	Daprodustat 4 mg once daily increased Hb over the first 4 weeks. Throughout the 24-week study, daprodustat achieved and maintained Hb within the target range and no new safety concerns were identified in hemodialysis patients not receiving erythropoiesis-stimulating agents	Tsubakihara et al. [84]
	271 HD-CKD patients received daprodustat or darbepoetin	40–52	Daprodustat was noninferior to darbepoetin alfa as measured by mean Hb Mean hepcidin levels decreased more at week 52 with daprodustat than with darbepoetin alfa Frequency of adverse events were generally similar between daprodustat and darbepoetin alfa.	Akizawa et al. [83]
Molidustat	161 ND-CKD patients ESA naïve received molidustat versus darbepoetin	52	Molidustat was noninferior to darbepoetin alfa as measured by mean Hb	Akizawa et al. [112]
	164 ND-CKD patients ESA treated received molidustat versus darbepoetin	52	Molidustat was noninferior to darbepoetin alfa as measured by mean Hb	Akizawa et al. [113]
	51 patients on peritoneal dialysis treated with molidustat	36	Molidustat maintained Hb levels in the prespecified range in more than half of the patients and was well tolerated.	Akizawa et al. [98]
	25 HD patients ESA-naive treated with molidustat	24	Treatment with dose-titrated molidustat for 24 weeks was well tolerated in Japanese patients undergoing hemodialysis, and no new safety signal was observed.	Akizawa et al. [114]
Enarodustat	216 subjects (102 ESA-naïve and 114 ESA-treated subjects	24	The efficacy of enarodustat was comparable to darbepoetin in anemic patients with CKD not requiring dialysis. No new safety concerns were identified compared with darbepoetin.	Akizawa et al. [115]
	132 ND-CKD and 136 HD patients	52	The long-term safety and efficacy of enarodustat were confirmed in Japanese anemic patients with chronic kidney disease.	Akizawa et al. [97]

NDD-CKD—nondialysis-dependent chronic kidney disease, HD-CKD—hemodialysis-chronic kidney disease, PD-CKD—peritoneal dialysis chronic kidney disease, MACE—major adverse cardiac events.

**Table 3:** Potential advantages and disadvantages of HIF-PHIs [5,104,105,106,107,108,109,110,111].

Potential Benefits	Potential Harm
- Improved iron utilization * - Reduced triglyceride, total cholesterol, and low-density lipoprotein levels * - Reduced peak EPO * - Potential of reducing mortality - Oral efficacy * - Protection from ischemic injuries - Effective for patients resistant to ESAs	Predisposition to pulmonary arterial hypertension * Tumor progression Thromboembolic disease Cyst growth-promoting effects in patients with autosomal dominant polycystic kidney disease (ADPKD) Proangiogenic effects in patients with vascular retinopathies such as diabetic retinopathy Vascular calcifications

* evidence in humans.

## Data Availability

Not applicable.
